# Increased regional homogeneity modulated by metacognitive training predicts therapeutic efficacy in patients with schizophrenia

**DOI:** 10.1007/s00406-020-01119-w

**Published:** 2020-03-25

**Authors:** Xiaoxiao Shan, Rongyuan Liao, Yangpan Ou, Pan Pan, Yudan Ding, Feng Liu, Jindong Chen, Jingping Zhao, Wenbin Guo, Yiqun He

**Affiliations:** 1 Department of Psychiatry, The Second Xiangya Hospital of Central South University, Changsha, 410011 Hunan China; 2 National Clinical Research Center on Mental Disorders, Changsha, 410011 Hunan China; 3 The Second Affiliated Hospital of Xinxiang Medical University, Xinxiang, Henan China; 4 Department of Radiology, Tianjin Medical University General Hospital, Tianjin, 300000 China

**Keywords:** Metacognitive training, Regional homogeneity, Support vector regression, Schizophrenia, Olanzapine

## Abstract

Previous studies have demonstrated the efficacy of metacognitive training (MCT) in schizophrenia. However, the underlying mechanisms related to therapeutic effect of MCT remain unknown. The present study explored the treatment effects of MCT on brain regional neural activity using regional homogeneity (ReHo) and whether these regions’ activities could predict individual treatment response in schizophrenia. Forty-one patients with schizophrenia and 20 healthy controls were scanned using resting-state functional magnetic resonance imaging. Patients were randomly divided into drug therapy (DT) and drug plus psychotherapy (DPP) groups. The DT group received only olanzapine treatment, whereas the DPP group received olanzapine and MCT for 8 weeks. The results revealed that ReHo in the right precuneus, left superior medial prefrontal cortex (MPFC), right parahippocampal gyrus and left rectus was significantly increased in the DPP group after 8 weeks of treatment. Patients in the DT group showed significantly increased ReHo in the left ventral MPFC/anterior cingulate cortex (ACC), left superior MPFC/middle frontal gyrus (MFG), left precuneus, right rectus and left MFG, and significantly decreased ReHo in the bilateral cerebellum VIII and left inferior occipital gyrus (IOG) after treatment. Support vector regression analyses showed that high ReHo levels at baseline in the right precuneus and left superior MPFC could predict symptomatic improvement of Positive and Negative Syndrome Scale (PANSS) after 8 weeks of DPP treatment. Moreover, high ReHo levels at baseline and alterations of ReHo in the left ventral MPFC/ACC could predict symptomatic improvement of PANSS after 8 weeks of DT treatment. This study suggests that MCT is associated with the modulation of ReHo in schizophrenia. ReHo in the right precuneus and left superior MPFC may predict individual therapeutic response for MCT in patients with schizophrenia.

**Electronic supplementary material:**

The online version of this article (10.1007/s00406-020-01119-w) contains supplementary material, which is available to authorized users.

## Introduction

Metacognitive deficits have been revealed in all phases of schizophrenia [1]. Psychological therapies, such as metacognitive training (MCT), may provide an additional treatment method for metacognitive deficits in patients with schizophrenia. MCT is a widely used novel group intervention for patients with schizophrenia and strengthens the self-awareness and insights of patients into these cognitive distortions to relieve the positive symptoms of psychosis, particularly paranoid ideation [2].

MCT has a significant effect on symptoms related to schizophrenia. Some randomized controlled studies revealed the efficacy of MCT in decreasing delusion severity [3], with sustained improvement even at 3 years follow-up [4]. Meta-analyses indicated that MCT could considerably improve positive symptoms, cognitive biases, and insight of patients with schizophrenia [5, 6]. Besides, Moritz et al. revealed significantly positive effects of MCT on social functioning, self-esteem, and quality of life at 3 years follow-up [4]. However, MCT-related neural effects on brain structure and function remain unknown.

Several studies have explored the relationship between metacognition capacity and brain function in schizophrenia [7, 8]. The medial prefrontal cortex (MPFC), posterior cingulate cortex (PCC), and precuneus, associated with cognitive functions, such as self-referential tasks, play a crucial role in metacognition capacity [9]. A positive correlation was observed between metacognitive ability and resting-state functional connectivity in the precuneus, PCC, and MPFC, suggesting that disrupted resting-state connectivity was associated with metacognitive dysfunction in psychosis [8]. Baird et al. [7] revealed a connection between metacognitive capacity relevant to memory retrieval and resting-state functional connectivity in the inferior parietal lobule structures/intraparietal sulcus, precuneus, and anterior MPFC. To our knowledge, few studies have explored MCT-related brain function alterations in patients with psychosis. Andreou et al. [10] found MCT-related task-positive network activity changes and effective connectivity between posterior inferior parietal cortex and other cortical regions in patients with delusion by region-of-interest analyses. Therefore, it is established that a relationship between MCT and brain function may exist in schizophrenia.

Although the majority of analysis techniques [i.e., independent component analysis (ICA), functional connectivity (FC), and graph theory] for resting-state fMRI data describe the function of brain network, the local brain activity cannot be addressed with these approaches. Regional homogeneity (ReHo) is a data-driven approach to determine how good the intra-cluster homogeneity is that other approaches cannot be decided [11, 12].

The ReHo approach can measure regional coherence of fMRI signal activity at low frequency (0.01–0.08 Hz), which reflects the local temporal homogeneity of the regional blood oxygen level-dependent (BOLD) signal, and describes the local connectivity of brain regions and synchronicity or similarity of time series of a given voxel with its immediate neighbors. Kendall’s coefficient concordance (KCC) is applied to measure the similarity or synchronization of the time series of a given voxel to those of its nearest neighbors in a voxel-wise way [11]. ReHo has some advantages [12]. First, unlike the coefficients of spontaneous low-frequency, integrated local correlation, and local functional connectivity density (lFCD), ReHo is a nonparametric data-driven method, and hence allows for exploring the temporally autocorrelated samples with non-normal distributions. ReHo has high robustness against spatial–tempo noise, which is beneficial for real resting-state fMRI time series [13–15]. Second, ReHo requires no prior knowledge about the abnormalities of brain structure or function. Therefore, it can be used as a potential data mining tool to examine high spatial resolution images of the brain. Finally, ReHo is relatively easy to compute and implements a graphical user interface in the software platform [16]. These benefits lay the foundation for the well application of ReHo in discovering brain function in healthy and disease states.

High ReHo may indicate neural hyperactivity in the brain regional area, and vice versa [17]. ReHo has been used to explore the abnormalities of regional functional synchronization in various psychiatric disorders, such as schizophrenia [18], depression [19], and individuals with prodromal psychosis [21].

Brain regional abnormality may be linked to clinical symptoms and prediction of treatment response. Several regions, including putamen, basal ganglia, and anterior cingulate cortex (ACC), are correlated with clinical outcome and involved in prediction of treatment response in schizophrenia [22–27]. For example, increased putamen volume was closely associated with positive symptomatic improvement assessed through a Positive and Negative Syndrome Scale (PANSS) reduction score from baseline to 6 weeks of treatment [22]. Another study reported that basal ganglia volume was associated with treatment response to antipsychotic medication evaluated by changes in Brief Psychiatric Rating Scale (BPRS) total scores from baseline (off medication) to 6 weeks of treatment [23]. The modulation of limbic circuitry might predict treatment response to antipsychotic medication, in which good therapeutic response was defined as > 10% improvement on the BPRS psychosis subscale scores after 6 weeks of treatment [24]. In addition, functional connectivity between ACC and other regions, such as putamen and anterior insula, was often reported to be related to prediction of treatment response for schizophrenia [28]. The baseline regional activity of the brain appeared to predict early response to treatment for schizophrenia [29]. However, previous studies on imaging predictors primarily focused on brain functional and structural changes after antipsychotic therapy. Few studies have reported imaging predictors induced by MCT therapy, which might help psychiatrists to make an advisable decision regarding the choice of therapy method before treatment initiation.

The prediction of psychosis based on neuroanatomical biomarkers is possible by applying multivariate pattern recognition approaches, such as support vector machine and support vector regression (SVR), which permit predictions to be conducted at the individual level. These techniques have been applied to discriminate patients with schizophrenia [30] and prodromal individuals [31] from healthy controls and predict treatment response of electroconvulsive treatment in schizophrenia and major depressive disorder (MDD) [32, 33].

In the present study, 41 patients with schizophrenia were recruited to explore the effect of drug plus psychotherapy (DPP) and drug therapy (DT) treatment on ReHo and its role in predicting individual therapeutic response. Clinical status and fMRI scans of inpatients with schizophrenia were obtained at two time points (baseline and 8 weeks of treatment). We hypothesized that MCT could enhance ReHo in patients with schizophrenia, particularly in the MPFC, PCC, and precuneus. Correlations between ReHo alterations and reductions in symptomatic severity were also expected to predict individual therapeutic response through SVR analyses.

## Materials and methods

### Participants

Forty-one patients with schizophrenia from the Second Affiliated Hospital of Xinxiang Medical University in China were recruited. Schizophrenia was diagnosed using the Structural Clinical Interview for Diagnostic and Statistical Manual of Mental Disorders, Fifth Edition (DSM-5). The total score of the PANSS was greater than 75, and the illness duration was not more than 5 years since the onset of the disease. The patients were right-handed and 18–38 years old. They were randomly allocated to the DPP and DT groups according to the random number list. Twenty patients were assigned to the DPP group, whereas 21 patients were in the DT group. The assessment scales were conducted by a clinical psychiatrist who was blinded to patient allocation. PANSS was used to evaluate symptomatic severity at baseline and 8 weeks of treatment. Cognitive function was assessed through the Measurement and Treatment Research to Improve Cognition in Schizophrenia Consensus Cognitive Battery, including the Brief Assessment of Cognition in Schizophrenia Symbol Coding Test (BACS-SC), Trail-Making Test, Part A, Brief Visuospatial Memory Test-Revised (BVMT-R), Hopkins Verbal Learning Test-Revised, Continuous Performance Test-Identical Pairs (CPT-IP), Neuropsychological Assessment Battery-Mazes, Wechsler Memory Scale Spatial Span, Category Fluency-Animal Naming Fluency (CF-ANF), and Mayer–Salovey–Caruso Emotional Intelligence Test (MSCEIT). These tests evaluated processing speed, working memory, attention/vigilance, reasoning, verbal learning, and problem solving.

Healthy controls unrelated to the patients were recruited from local community through advertisement. Age, years of education, and sex ratio of the patients and healthy controls were matched. The Structured Clinical Interview for DSM-IV, non-patient version, was applied to screen healthy controls. Healthy controls were excluded when they suffered from any medical and neurological illness, psychosis symptoms, and substance abuse. They were also ruled out if they have a first-degree relative with a history of psychiatric disorders.

The exclusion criteria for all subjects were as follows: any physical illness, such as cardiovascular, liver, and kidney diseases; any current or past neuropsychiatric disorders; any traumatic brain injury; seizures; serious impulsive behavior; drug or alcohol addiction; a history of electroconvulsive and olanzapine therapies that were ineffective or tolerable; contraindications for MRI; and pregnancy.

The study was approved by the ethics committee of the Second Affiliated Hospital of Xinxiang Medical University. The study was conducted in accordance with the Helsinki Declaration [34]. After a complete explanation, all subjects provided their written informed consent.

### Intervention

Olanzapine dosage increased within the first 2 weeks as clinically appropriate and remained unchanged until the last fMRI scan. The mean doses were 21.58 and 20.50 mg/day in the DPP and DT groups, respectively. The use of other antipsychotic medications was not allowed. On the basis of olanzapine therapy, the DPP group received MCT from a psychiatrist who had more than 1 year of experience with MCT. The DT group received a non-specific therapeutic program including some recreational activities as implemented in the Second Affiliated Hospital of Xinxiang Medical University. The total duration of the program and duration of the sessions and the frequency were matched to the MCT program.

MCT consisted of eight sessions guided by a trained clinical psychiatrist. The details of MCT processes are provided in the Supplemental Methods.

### Image acquisition and processing

A 3.0T Siemens scanner (Germany, Magnet Verio TimMR) was used to scan the patients at baseline (the first day after admission) and after 8 weeks of treatment. Healthy controls were scanned only once to determine brain regions with abnormal ReHo. Scanner parameters were as follows: repetition time/echo time = 2000/30 ms, 33 axial slices, 64 × 64 matrix, 90° flip angle, 22 cm field of view, 4 mm section thickness, 0.6 mm slice gap, and 240 volumes. All participants were required to lie still on the scanner with their eyes closed.

Data were preprocessed using the Data Processing Assistant for Resting-State fMRI (DPABI, versions 4.2) software. The first ten images were excluded from analysis due to the instability of the initial MRI signal and for the individuals to adapt to circumstances. Subjects with over 2 mm maximal translation in the *x*, *y*, or *z* axis and 2° maximal rotation in each axis were ruled out after slice timing and head motion correction. The imaging data were then spatially normalized to a conventional Montreal Neurological Institute (MNI) EPI template and resampled to 3 mm × 3 mm × 3 mm. The follow-up images of the patients were coregistered with baseline images before normalization. Finally, the data were temporally band-pass-filtered (0.01–0.08 Hz) and linearly detrended to reduce the effect of physiological high-frequency noise and low-frequency drifts. Several covariates, including signal from a ventricular region of interest, signal from a region centered in the white matter, and Friston-24 head motion parameters obtained via rigid body correction, were removed. The global signal was not removed. Besides, mean framewise displacement (FD) was used to solve the residual effects of motion as a covariate in group analyses. Scrubbing was also used as an aggressive head motion control strategy (removing time points with FD > 0.2 mm).

### ReHo analysis

The REST software (versions 1.8) (https://resting-fmri.sourceforge.net) was applied for the ReHo analysis. The cluster size for ReHo was 27 (one center voxel plus 26 nearest neighbors). The ReHo maps of each subject were obtained by calculating the KCC of the time series of a given voxel with those of its nearest neighbors. The KCC among each voxel was then divided by the mean KCC of the entire brain to normalize the ReHo maps. The generated ReHo maps were spatially smoothed with Gaussian kernel of 4 mm full-width at half maximum.

### Statistical analysis

Demographic and clinical characteristics were compared by Kruskal–Wallis test, Mann–Whitney *U* test or Chi-square test when necessary using Statistical Product and Service Solutions (SPSS, versions 20.0).

Two-sample *t* tests were performed to compare group differences between all patients at baseline and controls on voxel-based ReHo maps with age and FD as covariates. Repeated analyses of covariance (ANCOVAs) were conducted to assess the interaction effects between time points and groups with age and FD as covariates. False discovery rate (FDR) was employed to correct for multiple comparisons at *P* < 0.05 through the REST software.

To evaluate treatment effect, the following formula was applied to calculate the reduction ratio (RR) of the PANSS total scores.

$${\text{RR}} = \left( {{\text{PANSS}}_{{{\text{total}}\_1}} - {\text{PANSS}}_{{{\text{total}}\_2}} } \right)/{\text{PANSS}}_{{{\text{total}}\_1}} .$$

PANSS_total_1_ referred to the PANSS total scores at baseline, whereas PANSS_total_2_ was the PANSS total scores after 8 weeks of treatment. Similar RRs were calculated for the PANSS positive and negative symptoms and general psychopathology subscale scores.

### Correlation analyses

After brain clusters with abnormal ReHo were identified, the average ReHo values from these clusters were extracted. The correlations between ReHo alterations and changes in PANSS scores/cognition parameter scores of patients after treatment were determined using Pearson’s correlation analyses with a threshold of *P* < 0.05 by SPSS (versions 20.0).

### Classification analysis by using SVR

SVR was applied to test the capability of the extracted ReHo values in abnormal brain regions in predicting treatment response by using the LIBSVM software package (https://www.csie.ntu.edu.tw/~cjlin/libsvm/) in MATLAB. SVR was performed for extracted ReHo values (including baseline levels and alterations of ReHo) and each symptomatic domain (PANSS total and positive symptoms, negative symptoms, and general symptom subscale scores). The description on algorithm used and training set for SVR is given as follows.

Discovering a multivariate regression function *f*(*x*) on the basis of *X* through a sample spectrum is the purpose of predicting a desired output feature. The SVR equation is clearly clarified in the literature [35, 36] and summed up as follows [37]:

$$\left( x \right) = a_{0} + \mathop \sum \limits_{ij = 1}^{N} \left( {a_{i} - a_{i*} } \right)\left\{ {{\o}\left( i \right) \cdot {\o}\left( j \right)} \right\} + b,$$

where *α*_*i*_ and *α*_i*_ are the Lagrange multipliers meeting demand 0 ≤ *α*_*i*_, *α*_i*_ ≤ *C*. *C* is a supplementary parameter that appointed the regularization constant or penalty error, which defines the trade-off between the model simplicity and the training errors. Parameters *a* and *C* are comprehensively described in the literature [36, 38]. The parameter b is the substitution of the regression function *f*(*x*). ε-Insensitive loss function is an extra required factor widely applied for extensive SVR applications. The ability to process linear and non-linear data through the kernel is a valuable feature of the SVR. In the prediction process, the validity of the optimal model is tested. To optimize the parameters of the SVR model, the cross-validation approach is used to execute the parameter search [35]. To identify better values for ε and *C*, the training set is split into four subsets of equal size and one subset is examined using the predictor train in the remaining three subsets. A grid search is conducted over a pre-defined parameter space. The model which has the highest prediction accuracy is employed (i.e., lowest cross-validation error).

## Results

### Demographic and clinical characteristics

A total of 41 patients with schizophrenia and 20 healthy controls were enrolled in the study. However, two patients with schizophrenia (one in the DPP group and one in the DT group) were excluded due to excessive head movement. Thirty-nine patients with schizophrenia (19 in the DPP group and 20 in the DT group) were included in the final analysis. No significant difference was observed in the age, years of education, and sex ratios in the three groups. The mean dosage of olanzapine did not differ between the DPP and DT groups (Table 1). There was significant difference in the positive symptoms subscale scores (*P* < 0.05) between the two patient groups at baseline. By contrast, no substantial differences were observed in the general and negative symptoms subscale scores between the two groups.

**Table 1:** Demographic characteristics of the subjects

	DPP(*n* = 19)	DT (*n* = 20)	Controls (*n* = 20)	H/χ^2^	*P* value
Sex (male/female)	12/7	15/5	14/6	0.648	0.723^a^
Age (years)	26.05 ± 5.81	22.75 ± 4.38	25.70 ± 4.90	5.667	0.059^b^
Years of education (years)	11.63 ± 3.75	10.65 ± 2.50	12.75 ± 2.95	4.120	0.127^b^
Dose of olanzapine (mg/day)	21.58 ± 3.75	20.50 ± 1.54		0.459	0.498^c^

*DPP* drug plus psychotherapy, *DT* drug therapy

^a^The *P* values for sex distribution were obtained by a Chi-square test

^b^The *P* values were obtained by Kruskal–Wallis tests

^c^The *P* values were obtained by a Mann–Whitney *U* test

### Improvement in clinical symptoms after 8 weeks of treatment

As shown in Table 2, ANOVAs showed strong and significant group × time interactions for both PANSS total and positive symptoms subscale scores. A significant, but modest interaction was found for PANSS general symptoms subscale scores. By contrast, the group × time interaction was not significant for PANSS negative symptoms subscale scores. Compared to the baseline scores, DPP and DT groups exhibited significant improvement in PANSS negative symptoms subscale, positive symptoms subscale, general symptoms subscale, and total scores, and cognitive function tests after 8 weeks of treatment (p ≤ 0.001). The PANSS positive symptoms subscale, general symptoms subscale, and total scores in the DPP group were considerably lower than those in the DT group after 8 weeks of treatment (9.63 ± 2.24 vs. 12.3 ± 3.85; 24.95 ± 4.08 vs. 29.2 ± 5.51; 46.64 ± 7.97 vs. 56.05 ± 12.08, respectively) (*P* < 0.05). Several cognitive functioning measures, including BACS-SC, HVLT-R, WMS-SS, and CF-ANF also showed significant group × time interactions. Furthermore, the BACS-SC and CF-ANF scores in the DPP group were substantially higher than those in the DT group (49.89 ± 7.10 vs. 44.25 ± 11.02; 19.53 ± 2.25 vs. 17.95 ± 2.26, respectively) (*P* < 0.05) (Table 2).

**Table 2:** Comparison of the clinical characteristics between the DPP group and the DT group at each time point

	Test statistic	Baseline			*Z*	*P*	*Z*^*a*^	8 weeks		*Z*	*P*	*Z*^*b*^
		*P*	DPP group	DT group				DPP group	DT group			
PANSS			104.84 ± 9.96	103.0 ± 10.79	− 0.394	0.708	− 3.898	46.64 ± 7.97	56.05 ± 12.08	− 2.169	0.03	− 3.922
Time	F = 829.876	0.000										
Group × time	F = 9.515	0.0038										
Positive			24.89 ± 3.09	22.80 ± 5.82	− 2.003	0.047	− 3.826	9.63 ± 2.24	12.3 ± 3.85	− 2.426	0.015	− 3.886
Time	F = 281.287	0.000										
Group × time	F = 9.615	0.0037										
Negative			27.16 ± 5.19	27.4 ± 5.42	0.000	1.0	− 3.825	12.05 ± 3.19	14.55 ± 5.12	− 1.27	0.241	− 3.929
Time	F = 221.783	0.000										
Group × time	F = 1.443	0.237										
General			52.79 ± 5.13	52.8 ± 5.11	− 0.01	0.989	− 3.828	24.95 ± 4.0	29.2 ± 5.51	− 2.439	0.014	− 3.921
Time	F = 980.166	0.000										
Group × time	F = 6.665	0.014										
TMT-A			50.45 ± 18.13	55.09 ± 22.11	− 0.72	0.496	− 3.823	32.90 ± 13.08	33.44 ± 11.54	− 0.267	0.792	− 3.92
Time	F = 68.759	0.000										
Group × time	F = 0.751	0.392										
BACS-SC			38.37 ± 7.28	37.85 ± 10.79	− 1.17	0.247	− 3.729	49.89 ± 7.10	44.25 ± 11.02	− 2.293	0.021	− 3.929
Time	F = 78.836	0.000										
Group × time	F = 6.447	0.015										
HVLT-R			15.63 ± 4.19	17.15 ± 3.79	− 1.03	0.309	− 3.831	22.84 ± 3.39	22.1 ± 4.09	− 0.339	0.749	− 3.84
Time	F = 173.030	0.000										
Group × time	F = 5.979	0.019										
WMS-SS			11.26 ± 2.1	11.95 ± 2.65	− 0.575	0.588	− 3.832	15.95 ± 3.08	15.1 ± 2.94	− 1.018	0.322	− 3.947
Time	F = 134.380	0.000										
Group × time	F = 5.154	0.029										
NAB-M			8.63 ± 4.78	9.35 ± 5.66	− 0.042	0.967	− 3.828	16.11 ± 6.67	15.95 ± 5.71	− 0.07	0.945	− 3.929
Time	F = 95.522	0.000										
Group × time	F = 0.368	0.548										
BVMT-R			17.0 ± 6.68	18.35 ± 6.62	− 0.451	0.667	− 3.828	26.84 ± 6.06	27.05 ± 5.81	− 0.183	0.857	− 3.924
Time	F = 127.686	0.000										
Group × time	F = 0.484	0.491										
CF-ANF			13.10 ± 3.40	13.65 ± 3,22	− 0.82	0.428	− 3.833	19.53 ± 2.25	17.95 ± 2.26	− 2.163	0.033	− 3.932
Time	F = 214.891	0.000										
Group × time	F = 8.411	0.006										
MSCIT			76.71 ± 9.01	79.04 ± 9.19	− 0.843	0.411	− 3.823	88.50 ± 13.25	90.75 ± 13.28	− 0.702	0.496	− 3.92
Time	F = 69.445	0.000										
Group × time	F = 0.001	0.979										
CPT-IP			1.13 ± 0.89	1.03 ± 0.56	− 0.098	0.923	− 3.823	1.97 ± 0.97	1.80 ± 0.54	− 0.759	0.461	− 3.92
Time	F = 72.904	0.000										
Group × time	F = 0.167	0.685										

*Z*: the comparison between the DPP group and the DT group using Wilcoxon test of two independent samples. *Z*^*a*^: the comparison from baseline to 8 weeks within the DPP group using paired samples Wilcoxon test. *Z*^*b*^: the comparison from baseline to 8 weeks within the DT group using paired samples Wilcoxon test

*DPP* drug plus psychotherapy, *DT* drug therapy, *TMT-A* Trail Making Test, part A, *BACS-SC* Brief Assessment of Cognition in Schizophrenia Symbol Coding Test, *HVLT-R* Hopkins Verbal Learning Test-Revised, *WMS-SS* Wechsler Memory Scale Spatial Span, *NAB-M* Neuropsychological Assessment Battery-Mazes, *BVMT-R* Brief Visuospatial Memory Test-Revised, *CF-ANF* Category Fluency-Animal Naming Fluency, *MSCIT* Mayer-Salovey-Caruso Emotional Intelligence Test, *CPT-IP* Continuous Performance Test-identical Pairs

### Two-sample t test results

**Fig. 1:**
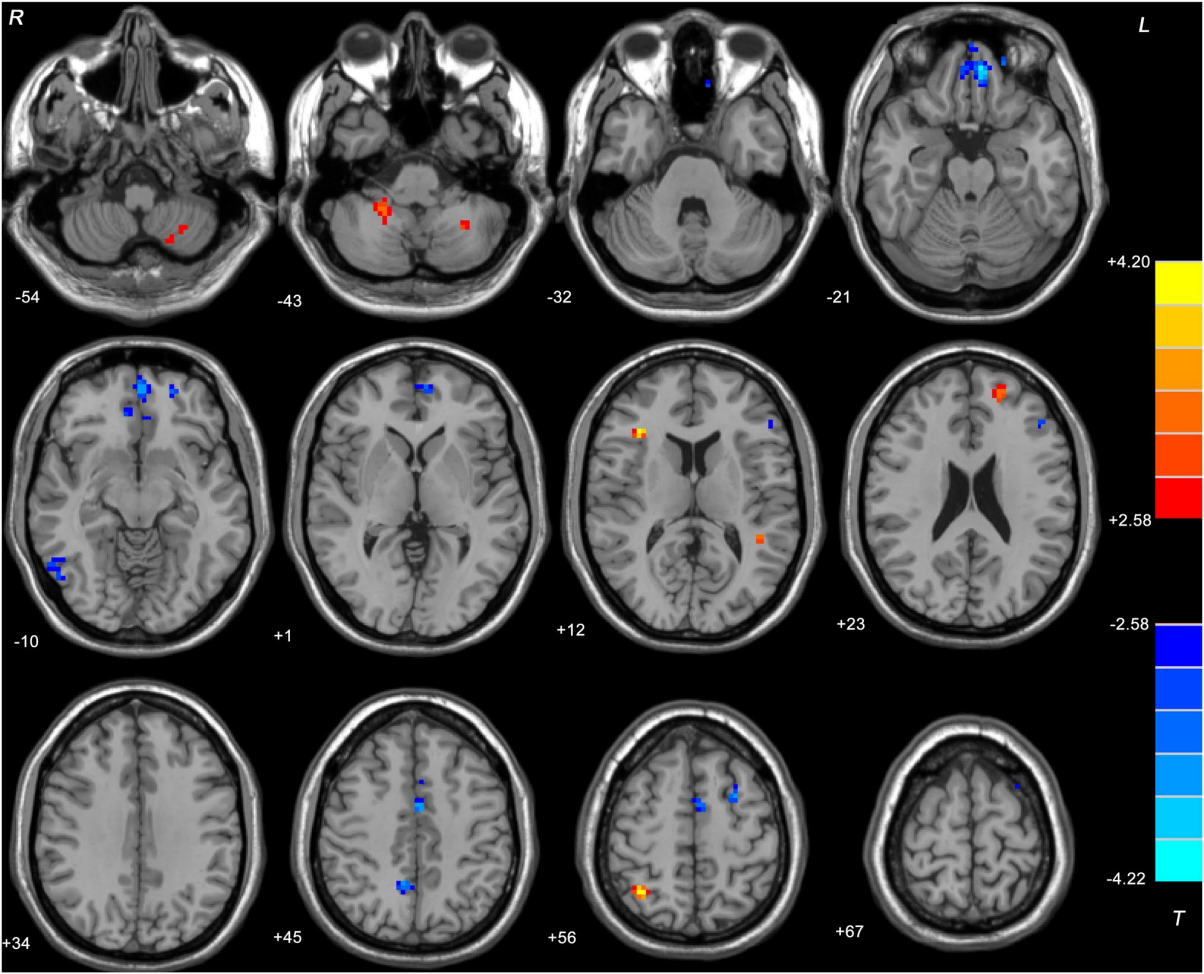
Brain regions with significant difference in ReHo between all patients at baseline and healthy controls. Brain regions with significant difference were observed in the bilateral ventral MPFC, left superior MPFC, right ITG, left triangular IFG, right precuneus, left SMA, left middle FG, left cerebellum VIII, left cerebellum X, right triangular IFG, left superior TG, left superior FG, and right superior parietal gyrus. The color bar represents the *t* values of the group analysis of ReHo. *ReHo* regional homogeneity, *MPFC* medial prefrontal cortex, *SMA* supplementary motor area, *ITG* inferior temporal gyrus, *IFG* inferior frontal gyrus

Patients (both DPP and DT groups) at baseline showed significantly decreased ReHo values in the bilateral ventral MPFC, left superior MPFC, right inferior temporal gyrus, left triangular inferior frontal gyrus (IFG), right precuneus, left supplementary motor area, and left MFG and increased ReHo values in the left cerebellum VIII, left cerebellum *X*, right triangular IFG, left superior temporal gyrus (TG), left superior FG, and right superior parietal gyrus compared with the controls (Table 3 and Fig. 1). There was no significant difference in ReHo between the two patient groups at baseline.

**Table 3:** Alterations of ReHo among patients (at baseline, after 8 weeks of treatment) and controls

Cluster location	Peak coordinate			Cluster (voxel)	*T* value
	*x*	*y*	*Z*		
Patients (both DPP and DT groups) at baseline versus controls					
Left cerebellum VIII	− 27	− 57	− 51	36	3.3466
Right cerebellum X	21	− 45	− 42	23	3.3674
Left superior MPFC	− 21	51	− 15	31	− 4.174
Bilateral ventral MPFC	− 6	48	− 24	202	− 4.221
Right ITG	54	− 69	− 12	32	− 3.1499
Left superior TG	− 45	− 42	15	26	3.7986
Right triangular IFG	36	27	12	26	3.9728
Left triangular IFG	− 48	33	18	31	− 4.0695
Left superior FG	− 18	54	21	26	3.4123
Right Precuneus	9	− 54	42	23	− 3.5559
Left SMA	0	0	45	56	− 3.617
Left middle FG	− 27	6	60	23	− 3.4196
Right superior Parietal gyrus	33	− 57	60	30	4.2025
DPP group versus DT group at baseline					
No cluster					
DPP group after 8 weeks versus at baseline					
Left superior MPFC	− 21	51	− 12	37	4.9128
Right precuneus	9	− 54	45	21	2.8237
Right Parahippocampal	30	− 21	− 27	21	3.7648
Left Rectus	0	48	− 24	20	2.9517
DT group after 8 weeks versus at baseline					
Left ventral MPFC/ACC	− 6	39	− 12	79	6.0404
Left superior MPFC/ MFG	− 30	36	36	34	3.241
Left precuneus	− 9	− 72	39	29	3.5342
Left cerebellum VIII	− 27	− 60	− 54	22	− 3.7028
Right cerebellum VIII	21	− 51	− 33	31	− 3.8874
Right rectus	9	48	− 21	29	3.3901
Left IOG	− 30	− 75	− 6	23	− 3.2013
Left MFG	− 30	6	60	21	2.8294

The significance level was set at *P* < 0.05 corrected by the false discovery rate (FDR) method for multiple comparisons with the REST software (age and FD as covariates)

*DPP* drug plus psychotherapy, *DT* drug therapy, *ReHo* regional homogeneity, *MPFC* medial prefrontal cortex, *IFG* inferior frontal gyrus, *ITG* inferior temporal gyrus, *SMA* supplementary motor area, *ACC* anterior cingulate cortex, *MFG* middle frontal gyrus, *IOG* inferior occipital gyrus, *FD* framewise displacement

### Repeated ANCOVA ReHo results

**Fig. 2:**
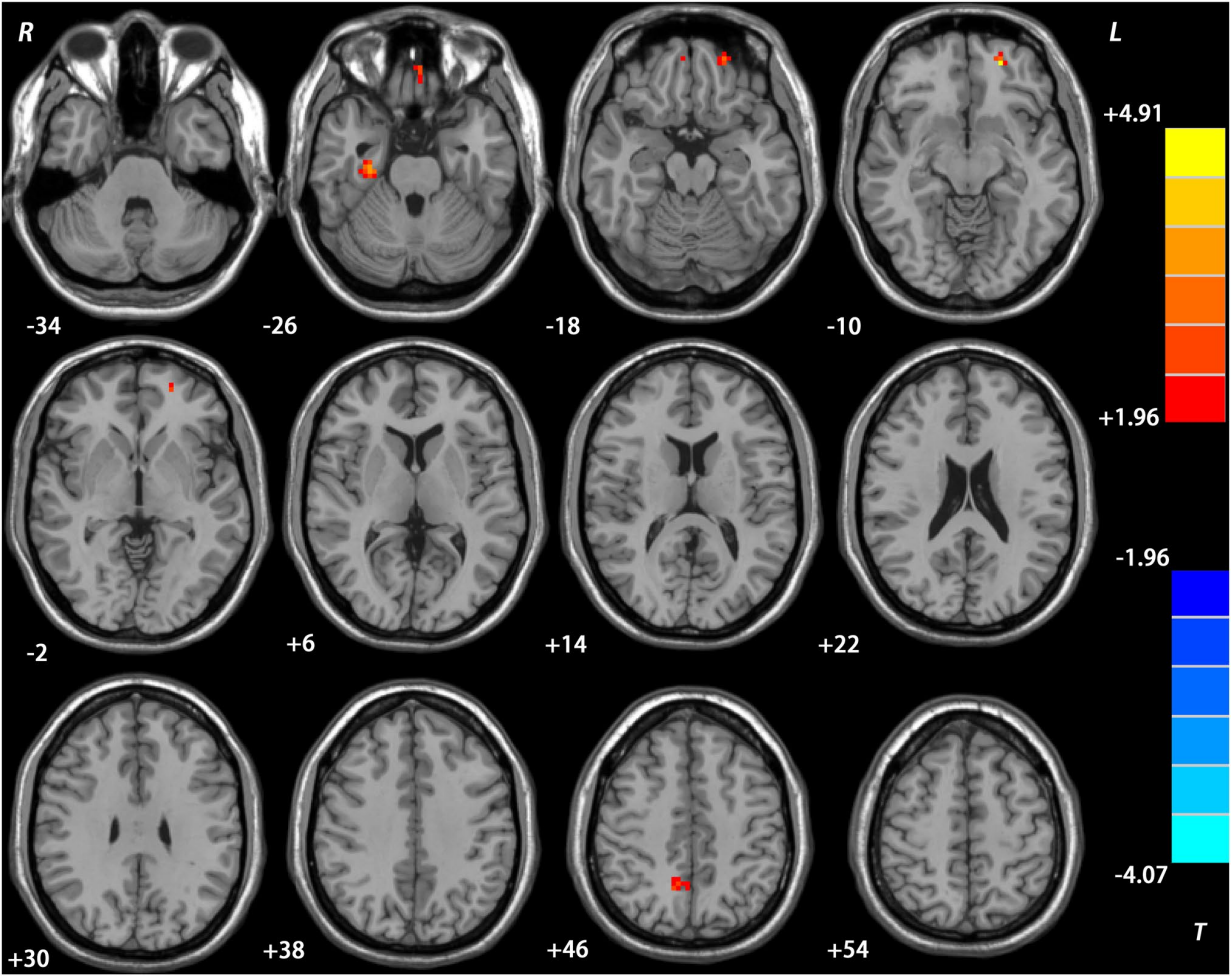
Treatment effects on ReHo in the DPP group. Brain regions with significant difference in ReHo were observed in the right precuneus, left superior MPFC, right parahippocampal gyrus, and left rectus after 8 weeks of treatment. The color bar represents the *t* values of the group analysis of ReHo. *DPP* drug plus psychotherapy, *ReHo* regional homogeneity, *MPFC* medial prefrontal cortex

**Fig. 3:**
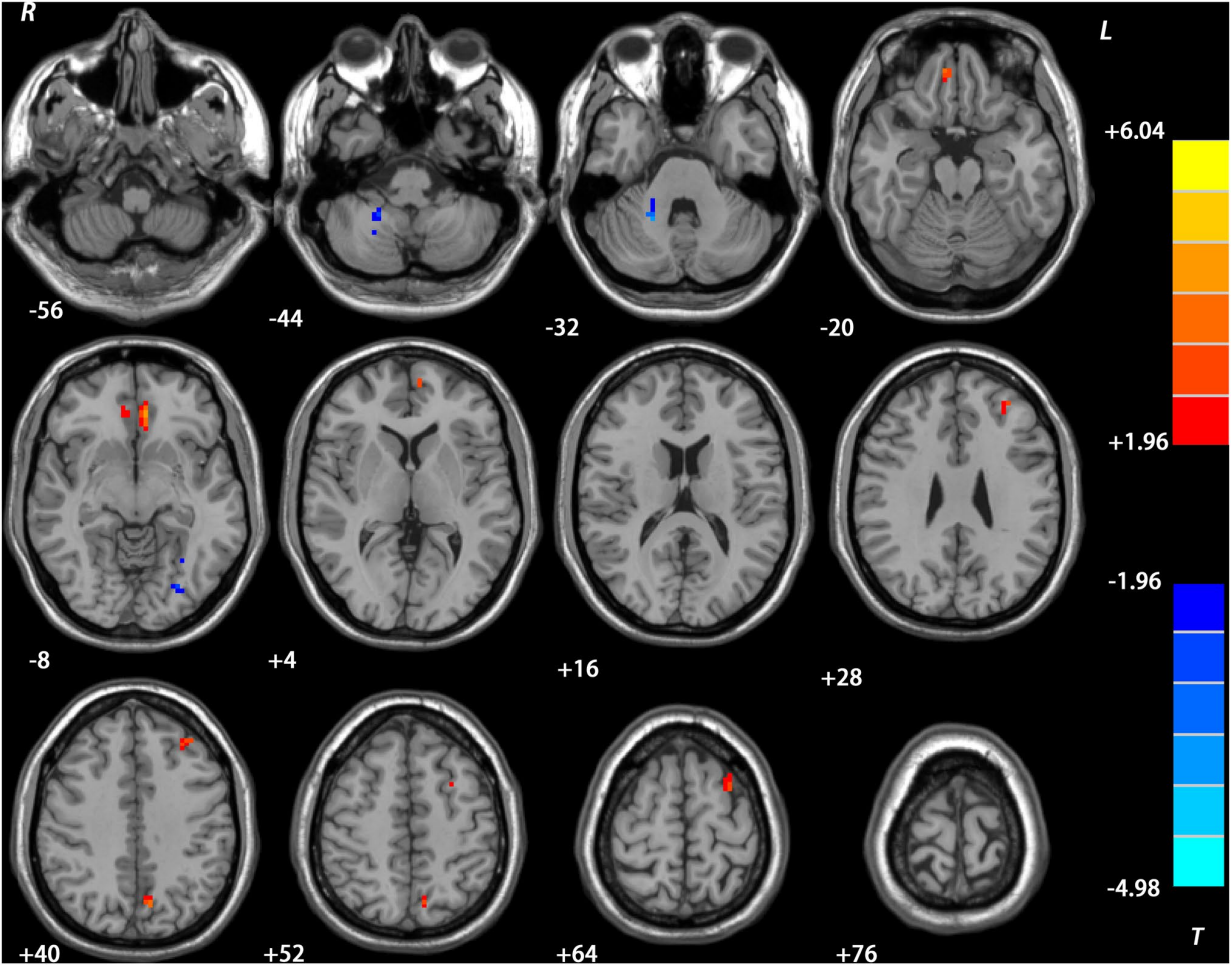
Treatment effects on ReHo in the DT group. Brain regions with significant difference in ReHo were observed in the left superior MPFC/ MFG, left ventral MPFC /ACC, left precuneus, right rectus, left MFG, left IOG and bilateral cerebellum VIII after treatment. The color bar represents the *t* values of the group analysis of ReHo. *DT* drug therapy, *ReHo* regional homogeneity, *MPFC* medial prefrontal cortex, *ACC* anterior cingulate cortex, *MFG* middle frontal gyrus, *IOG* inferior occipital gyrus

Compared with the baseline data, the DPP group showed significantly increased ReHo in the right precuneus, left superior MPFC, right parahippocampal gyrus, and left rectus after 8 weeks of treatment (Table 3, Fig. 2), whereas the DT group exhibited significantly increased ReHo value in the left ventral MPFC/ACC, left superior MPFC/middle frontal gyrus (MFG), left precuneus, right rectus, and left MFG, and significantly decreased ReHo value in the left inferior occipital gyrus (IOG) and bilateral cerebellum VIII after treatment (Table 3, Fig. 3). Besides, there was no significant group × time interactions on ReHo.

### Correlation results

**Fig. 4:**
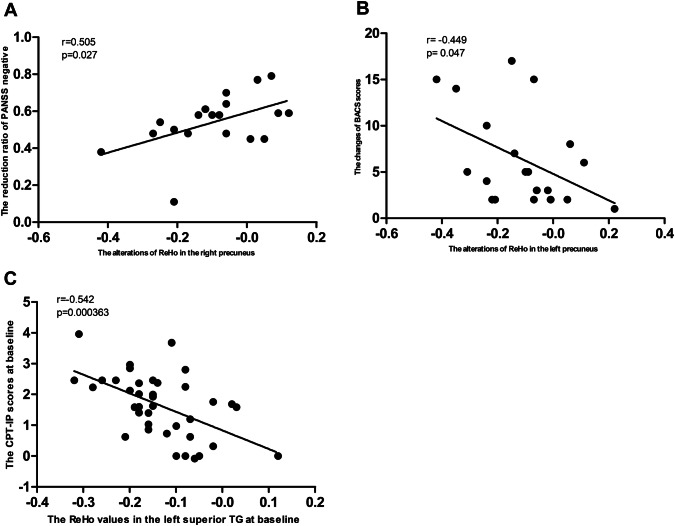
Correlations between ReHo and PANSS scores/cognitive scores in the patients. A: correlations between alterations of ReHo in the right precuneus and changes in PANSS negative symptoms subscale scores in the DPP group after 8 weeks of treatment. B: correlations between alterations of ReHo in the left precuneus and changes in BACS scores in the DT group after 8 weeks of treatment. C: correlations between increased ReHo in the left superior TG and CPTIP scores in all patients (both DPP and DT groups) at baseline. *DPP* drug plus psychotherapy, *DT* drug therapy, *ReHo* regional homogeneity, *PANSS* Positive and Negative Syndrome Scale; *TG* temporal gyrus, *BACS* Brief Assessment of Cognition in Schizophrenia, *CPTIP* Continuous Performance Test-identical Pairs

Baseline ReHo values in the left superior TG were negatively correlated with the CPT-IP (*r* = − 0.542, *P* = 0.000363, uncorrected). The alterations of ReHo values in the right precuneus were positively correlated with changes in PANSS negative symptoms in the DPP group (*r* = 0.505, *P* = 0.027, uncorrected), whereas no correlations were found between alterations in ReHo values and changes in PANSS scores in the DT group. The alterations of ReHo values in the left precuneus were negatively correlated with changes of BACS in the DT group (*r* = − 0.449, *P* = 0.047, uncorrected) (Fig. 4). However, these correlations were not significant after Bonferroni correction (*P* < 0.05/338 = 0.0001479).

### SVR analyses

To test whether the extracted ReHo in the brain regions could predict therapeutic response after DPP or DT treatment, SVR analyses were conducted.

**Fig. 5:**
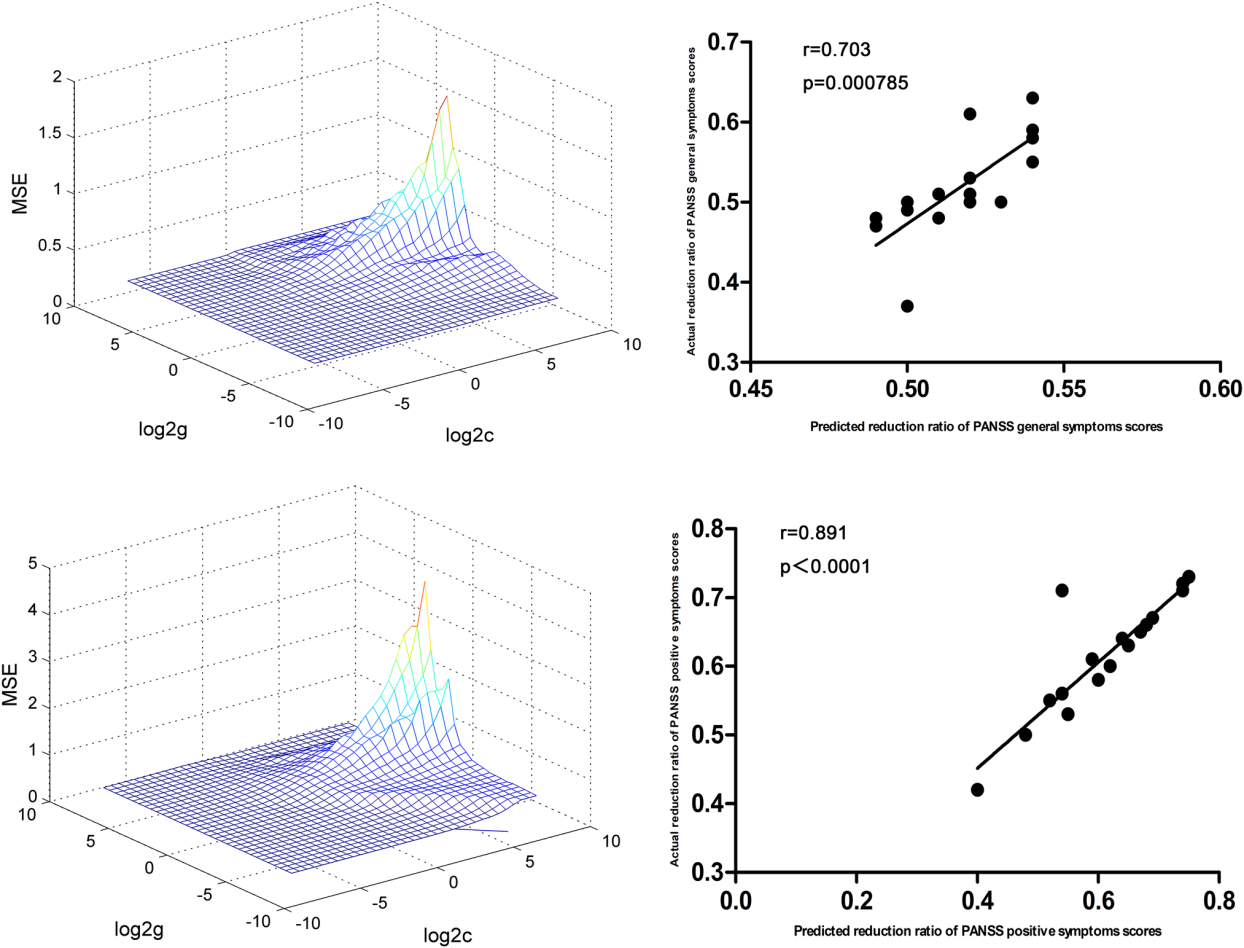
SVR results suggested that high ReHo levels at baseline in the left superior MPFC could predict therapeutic response in the DPP group. Left: SVR parameter selection results (3D visualization); Right: The positive correlations between predicted and actual RR of the PANSS positive symptoms subscale scores(*r* = 0.891, *P* <  0.0001), general symptoms subscale scores (*r* = 0.703, *P* = 0.000785) of individual patients after 8 weeks of DPP treatment. *DPP* drug plus psychotherapy, *ReHo* regional homogeneity, *SVR* support vector regression, *MPFC* medial prefrontal cortex, *PANSS* Positive and Negative Syndrome Scale, *RR* reduction ratio

**Fig. 6:**
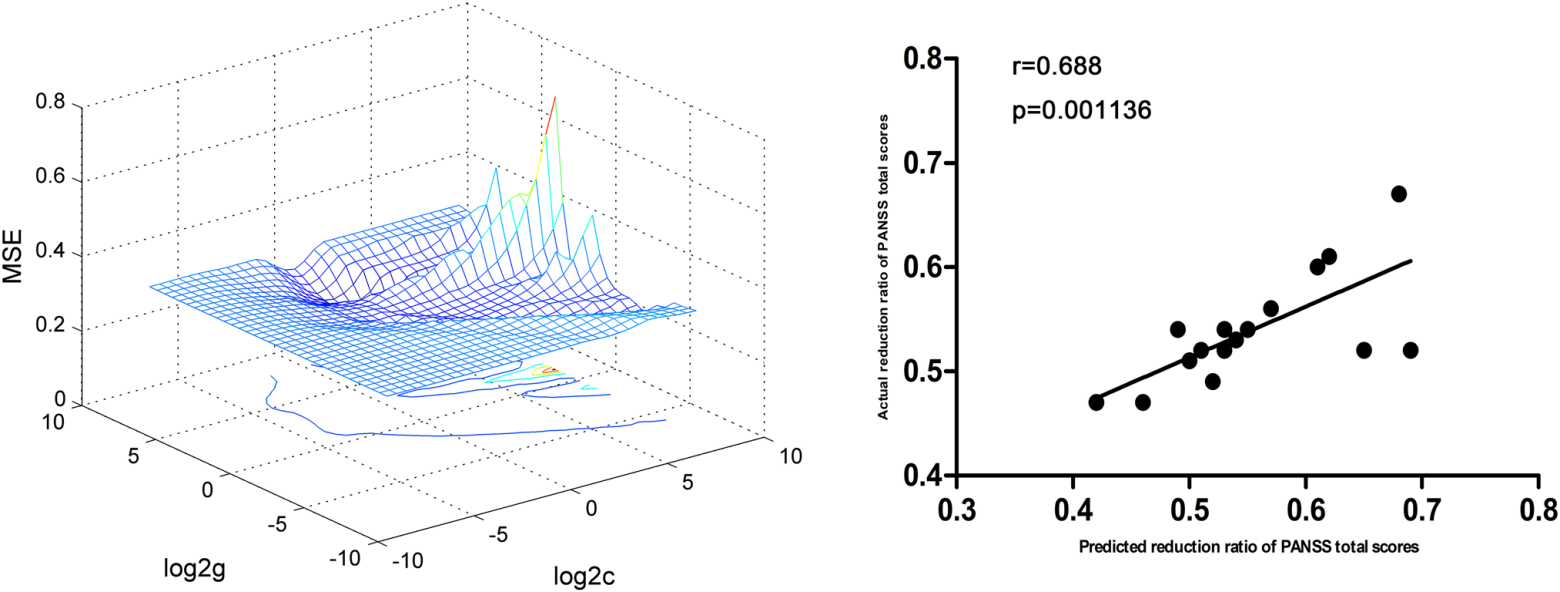
SVR results suggested that high ReHo levels at baseline in the right precuneus could predict therapeutic response in the DPP group. Left: SVR parameter selection results (3D visualization); right: the positive correlations between predicted and actual RR of the PANSS total scores (*r* = 0.688, *P* = 0.001136) of individual patients after 8 weeks of DPP treatment. *DPP* drug plus psychotherapy, *ReHo* regional homogeneity, *SVR* support vector regression, *PANSS* Positive and Negative Syndrome Scale, *RR* reduction ratio

At *P* < 0.05/40 = 0.00125 level (Bonferroni correction), there were significantly positive correlations between baseline ReHo values in the left superior MPFC and RR of the PANSS positive symptoms subscale scores (*r* = 0.891, *P* < 0.0001) and general symptoms subscale scores (*r* = 0.703, *P* = 0.000785) in the DPP group (Fig. 5). Significantly positive correlations between baseline ReHo values in the right precuneus and RR of the PANSS total scores (*r* = 0.688, *P* = 0.001136) were observed in the DPP group (Fig. 6).

For the DT group, SVR results showed significantly positive correlations between baseline ReHo values in the left ventral MPFC/ACC and RR of PANSS scores. Furthermore, positive correlations between alterations of ReHo values in the left ventral MPFC/ACC, and RR of PANSS scores were also observed. The details of related data are provided in Supplemental Figures.

## Discussion

To our knowledge, this longitudinal study is the first to explore ReHo with MCT and olanzapine therapy in schizophrenia. The results revealed that ReHo values in the left superior MPFC, right precuneus, right parahippocampal gyrus, and left rectus were significantly increased within the DPP group after 8 weeks of treatment. By contrast, significantly increased ReHo values were observed in the left superior MPFC/MFG, left ventral MPFC/ACC, left precuneus, right rectus, and left MFG, and significantly decreased ReHo values in the left IOG and bilateral cerebellum VIII within the DT group after 8 weeks of treatment. Furthermore, SVR results showed that baseline ReHo values in the right precuneus and left superior MPFC could predict symptomatic improvement of PANSS after 8 weeks of DPP treatment.

Previous studies have revealed normalization and denormalization of the fMRI signal in schizophrenia [39–41]. Our findings showed significantly increased ReHo in the right precuneus of patients in the DPP group, which reflected local synchronization enhancement of spontaneous neural activities in this region. DPP treatment increased ReHo in the right precuneus, whereas decreased ReHo at baseline became almost normal after 8 weeks of treatment (normalization), although the ReHo values remain lower than those of the controls. By contrast, increased ReHo values in the left precuneus, not the right precuneus, were found in the DT group. Therefore, the effects of normalization on ReHo in the right precuneus might be associated with MCT treatment. Besides, there is no significant group × time interaction on ReHo in the present study. The lack of a significant group × time interaction does not imply that ReHo is a stable imaging marker. There are significant changes in ReHo in both groups over time. Furthermore, the stability of a marker cannot be tested by measures obtained before and after an intervention. Such measures can only compare response to the intervention. The fact that there are significant changes after the intervention compared with baseline does imply that there is a response to the intervention. The lack of a significant group × time interaction (and of a significant difference of groups in follow-up) implies that the changes in both groups are to be attributed to the common part of the intervention, i.e., there is no significant effect of MCT on ReHo added to the effect of olanzapine treatment. While different changes in groups might be interpreted as hints of an MCT effect, such an interpretation must be very cautious because of the above-mentioned arguments.

The volume, function, and connectivity of the frontal polar region and precuneus have associations with metacognitive ability. The precuneus is an important area of the default-mode network (DMN), which is involved in episodic memory retrieval, visuospatial imagery, self-referential tasks, consciousness, and cognitive functions such as spatial navigation [9, 42]. The precuneus activation is connected with the vividness of judgments in the time of episodic memory retrieval [43]. Compared with healthy controls, patients with schizophrenia had less activation in the precuneus when performing self- and other reflectivity tasks [44]. The activity of the right precuneus reflects detailed representations in the subjective contents in time of vivid relative to non-vivid recollection [45]. Although initial functional MRI studies have shown that the anterior PFC is associated with visual metacognition [46, 47], recent studies have revealed the important role of the precuneus in memory metacognition [7, 48], such as episodic memory retrieval [49]. For example, relationship between memory metacognitive efficiency and resting-state functional connectivity was revealed in the precuneus and MPFC [7]. Structural MRI studies also showed the correlation between gray matter density in the precuneus and memory metacognitive efficiency [48]. Hence, it seems reasonable that this region plays a role in performing higher-order monitoring of memory patterns from an anatomical standpoint. Moreover, McCurdy et al. indicated that the precuneus volume was weakly correlated with visual metacognitive efficiency, suggesting that the precuneus might be involved in a common mechanism for memory and visual metacognition [48]. Our study showed increased ReHo in the right precuneus after DPP treatment, which was different from the ReHo alterations after DT treatment. Together with the above-mentioned findings, our study further supported the important role of precuneus in metacognitive ability, suggesting the involvement of the precuneus in the potential neurological mechanism of modulating ReHo for MCT.

The frontal cortex has well‐established effects on the mnemonic activities, cognitive processing, and general monitoring of external and internal environments. Previous studies exploring individual metacognitive ability showed that the volume, function, and connectivity of anterior PFC were bases of introspective accuracy. Initial functional MRI studies have shown that the frontal lobe, especially anterior PFC, is associated with visual metacognition [46]. Indirect evidence suggests that metacognitive mechanisms in the anterior PFC may be universal for different visual tasks [50]. In addition, frontal hypoactivity and hypoconnectivity were revealed in untreated patients with schizophrenia [40, 51]. These abnormal conditions might reflect reduced blood flow or glucose metabolism in the frontal region. Furthermore, reduced glucose metabolism in the prefrontal regions is associated with negative symptoms [51] and cognitive dysfunction [52]. Our result showed increased ReHo in the left superior MPFC after 8 weeks of DPP treatment, where decreased ReHo was observed in this region at baseline. Increased ReHo in the left superior MPFC may reflect a temporal reorganization of regional neural activity and is related to increased glucose metabolism or blood flow in the MPFC region after treatment. These correlations may be a beneficial effect of DPP treatment considering that neurons in this region are active in a synchronous manner. Furthermore, increased ReHo in the left superior MPFC was also found in the DT group after treatment, and previous studies reported increased activity and connectivity in the left superior MPFC after olanzapine treatment [39, 41]. Hence, the observed changes in the left superior MPFC might be due to the effects of antipsychotic therapy.

DPP increased ReHo in the right parahippocampal gyrus after 8 weeks of treatment. No abnormality in ReHo was observed in this region before treatment. Abnormal activity and connectivity of the parahippocampal gyrus have been revealed in schizophrenia [53, 54]. These abnormal conditions might expose decreased glucose metabolism or blood flow in the parahippocampal areas. Moreover, meta-analysis revealed that parahippocampal gyrus was related to functional outcome in patients with schizophrenia, including social functioning and quality of life [55]. Although parahippocampal hypoconnectivity was not observed in the patients at baseline, increased ReHo in the parahippocampal gyrus might be associated with a temporal reorganization of region neural activity and a temporal integration of activity across the brain networks after DPP treatment.

Further SVR results showed that high ReHo levels at baseline of ReHo in the right precuneus and left superior MPFC could predict symptomatic improvement of PANSS after 8 weeks of DPP treatment. Meanwhile, high ReHo levels at baseline and alterations of ReHo in the left ventral MPFC/ACC could predict symptomatic improvement of PANSS after 8 weeks of DT treatment. Yuan et al. found that the precuneus, dorsal MPFC, and frontal orbital cortex were related to predicting long-term clinical outcome in post-traumatic stress disorder through fMRI parameters [56]. The left postcentral gyrus and precuneus were associated with the prediction of electroconvulsive therapy response [57]. Masuda et al. found that the hemodynamic activities in the frontotemporal cortex could predict response to selective serotonin reuptake inhibitor treatment in MDD [58]. Consistent with these studies, our findings that increased ReHo in the right precuneus and left superior MPFC could predict clinical treatment response highlight the importance of these two regions in MCT and contribute to interpret clinical symptomatic improvement in psychiatric disorders.

While improvement in clinical symptoms was observed in the two patient groups, the DPP group appeared to have a stronger influence on PANSS positive symptoms subscale scores. The PANSS positive symptoms subscale scores in the DPP group were significantly higher than those in the DT group at baseline. However, after 8 weeks of treatment, the DPP group scored significantly lower in the PANSS positive symptoms subscale than those in the DT group, suggesting DPP treatment had greater improvement in positive symptoms than DT treatment. Furthermore, the BACS-SC and CF-ANF scores in the DPP group were substantially higher than those in the DT group after 8 weeks of treatment, suggesting that MCT exhibited a beneficial effect on clinical symptoms and some cognitive functions, including memory and attention. These results are consistent with several previous findings, which showed that MCT was associated with the improvement in positive symptoms, especially delusions and multiple neurocognitive components [6, 59, 60]. There were some possibilities for the result. First, clinical symptoms of the patients were improved comprehensively after treatment, and improvement of positive symptoms in the DPP group was more obvious than that in the DT group, suggesting that MCT was more effective to positive symptoms in the patients. Second, both patient groups exhibited significant improvement in PANSS negative symptoms subscale scores after 8 weeks of treatment compared to the baseline data. However, improvement in PANSS negative symptoms subscale scores in the DPP group is not significantly different from that in DT group. Previous studies on the longitudinal course of negative symptoms have revealed that negative symptoms gradually became obvious over time and eventually dominated the clinical presentation [61, 62]. All patients received olanzapine treatment. Hence, combined with the findings from previous studies, the improvement in negative symptoms in the present study might be due to the effect of medication use and not to the time.

After Bonferroni correction, there are no significant correlations between abnormal ReHo values and PANSS scores/cognition parameters in the patients at baseline and between ReHo alterations and changes in PANSS scores/cognition parameter scores in the patients after treatment. Thus, ReHo in these regions could not be used as a quantitative marker for evaluation of clinical symptom severity, although it could help locate dysfunctional brain regions.

Apart from its small sample size, the present study has several limitations. First, MCT was conducted for only 8 weeks (1 session a week) in the study, including half of the whole program. The insufficient treatment might reduce treatment efficacy of MCT in the present study. Second, Bonferroni correction used in the present study is a little strong (at *P* < 0.05/338 = 0.0001479 level) and false negative correlation results may exist. Therefore, it is important to cautiously interpret the non-significant (when corrected) findings.

In conclusion, the present study is the first to evaluate ReHo associated with MCT in patients with schizophrenia. MCT may enhance ReHo in the right precuneus, left superior MPFC, right parahippocampal gyrus, and left rectus in schizophrenia. Increased ReHo in the right precuneus and left superior MPFC may predict individual therapeutic response in clinical symptoms to MCT-related therapy in patients with schizophrenia. This study suggests that MCT is associated with the modulation of ReHo in schizophrenia. ReHo in the right precuneus and left superior MPFC may predict individual therapeutic response for MCT in patients with schizophrenia.

## Electronic supplementary material

Below is the link to the electronic supplementary material.

Supplementary file1 (DOCX 635 kb)
