# Supplementary material for: Increased regional homogeneity modulated by metacognitive training predicts therapeutic efficacy in patients with schizophrenia
Source: Eur Arch Psychiatry Clin Neurosci. 2020 Mar 25;271(4):783–98. doi: 10.1007/s00406-020-01119-w (PMC8119286; doi:10.1007/s00406-020-01119-w)
Supplement: Supplementary file 1 — Supplementary file1 (DOCX 635 kb) [file 406_2020_1119_MOESM1_ESM.docx]

**Supplemental Methods**

**Metacognitive training (MCT)**

MCT was performed in the drug plus psychotherapy (DPP) group. Each session of MCT lasted for approximately 45–60 min. In each session, patients with schizophrenia were required to familiarize themselves with a particular bias through a comprehensive PowerPoint presentation. Daily life examples were then used to demonstrate the correlation between this particular cognitive bias and psychotic experiences. Afterward, group exercises were conducted aimed at tackling the biases. The patients were then given assignments and leaflets with the information in the form of exercises. The training program consisted of a cycle with eight modules (free download at [www.uke.de/mct](http://www.uke.de/mct)). Module training was executed once a week. Approximately 3–10 patients were included in the treatment group. The titles of the eight modules were as follows: attribution-blaming and taking credit (emphasizing the shortcomings of monocausal inferences), jumping to conclusions I (patients are advised not to make hasty decisions), changing beliefs (teaching patients to be flexible and stay open to different interpretations), to empathize 1 (guiding patients to be aware of  multiple social cues before inferring another person's mental state), memory (patients learn to refrain from strong judgments and overconfidence in false memories), to empathize II, jumping to conclusions II, and self-esteem and mood (CBT-based techniques are used to boost self-esteem). MCT can help alter patients’ current problem-solving repertoire. The main purpose of MCT for patients with schizophrenia is to enhance the patients’ consciousness of their cognitive distortions in an enjoyable and cultivate manner.

**Supplemental Figures**

**Figure S1.** SVR results suggested that high ReHo levels at baseline in the left ventral MPFC/ACC could predict therapeutic response in DT group. Left: SVR parameter selection results (3D visualization); Right: The positive correlations between predicted and actual RR of the PANSS negative symptoms subscale scores (r=0.882, p＜0.0001), general symptoms subscale scores (r=0.702,p=0.000567) and total scores (r=0.729, p= 0.000268) of individual patients after eight weeks DT treatment. DT=drug therapy; ReHo= regional homogeneity; MPFC= medial prefrontal cortex; ACC= anterior cingulate cortex; SVR = support vector regression; PANSS = Positive and Negative Syndrome Scale; RR= reduction ratio.

**Figure S2.** SVR results suggested that alterations of ReHo values in the left ventral MPFC/ACC could predict therapeutic response in DT group. Left: SVR parameter selection results (3D visualization); Right: The positive correlation between predicted and actual RR of the PANSS positive symptoms subscale scores (r=0.793, p=0.000030), negative symptoms subscale scores (r=0.872, p=0.000001) of individual patients after eight weeks DT treatment. DT=drug therapy; ReHo= regional homogeneity; MPFC= medial prefrontal cortex; ACC= anterior cingulate cortex; SVR = support vector regression; PANSS = Positive and Negative Syndrome Scale; RR= reduction ratio.

**Figure S1**


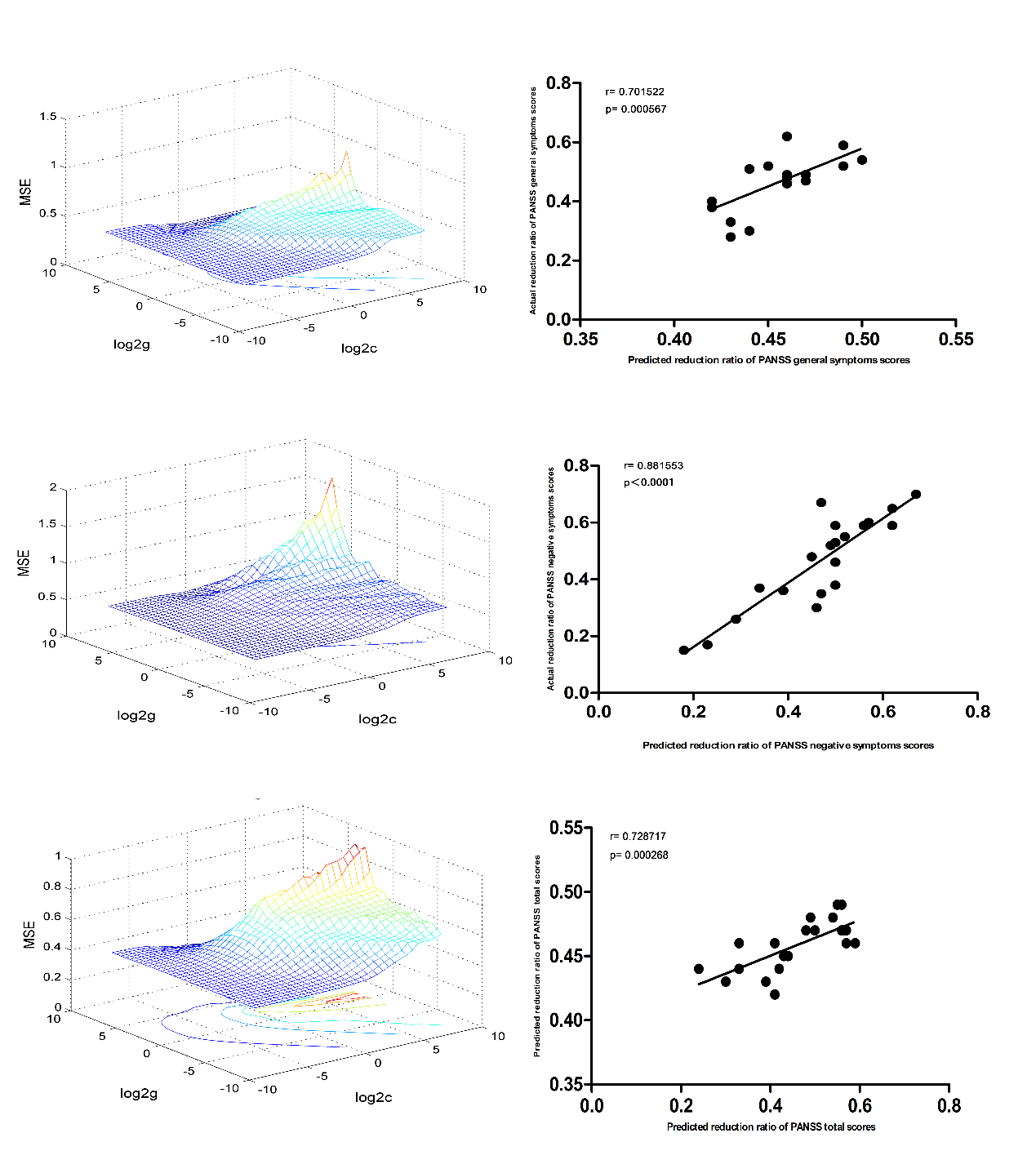


**Figure S2**

**
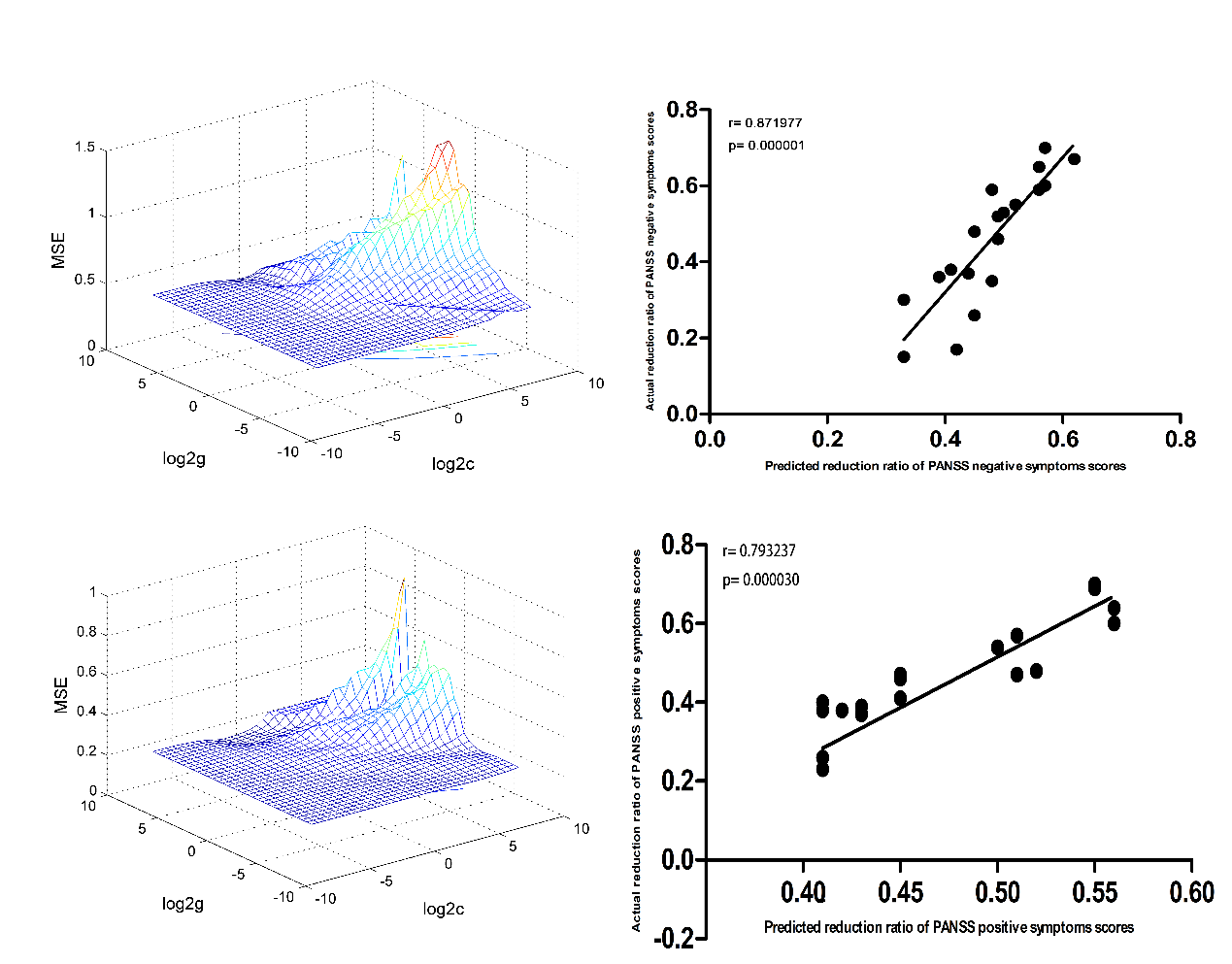
**
